# *Emblica officinalis* Garten fruits extract ameliorates reproductive injury and oxidative testicular toxicity induced by chlorpyrifos in male rats

**DOI:** 10.1186/2193-1801-2-541

**Published:** 2013-10-17

**Authors:** Abir Lal Dutta, Chitta Ranjan Sahu

**Affiliations:** Cell and Developmental Biology Laboratory, Department of Zoology, University of Kalyani, Kalyani, Nadia, West Bengal pin- 741235 India

**Keywords:** Organophosphate pesticides, Chlorpyrifos, *Emblica officinalis*, Testicular toxicity, Reproductive injury, Oxidative stress, Natural medicine

## Abstract

Organophosphate pesticides have destroying properties on male reproduction and chlorpyrifos adversely affects the male reproductive system. *Emblica offcinalis* Garten plays a vital role to challenge many diseases in human body. We investigated the induction of oxidative stress in the male reproductive system of adult rats (Wistar Strain) exposed to widely used organophosphate pesticide, Chlorpyrifos, and tried to establish the ameliorative properties of *Emblica officinalis* Garten with respect to reproductive reconstruction in them. Rats were divided into 2 groups, control group and experimental group, and the experimental group was divided into 3 groups (G1-G3). All the groups had 5 rats each. Control group received water, experimental group, G1, received 20 mg/kg bw/day *Emblica officinalis* Garten, G2 received 12 mg/kg bw/day chlorpyrifos and G3 received 12 mg chlorpyrifos with 20 mg *Emblica officinalis* Garten /kg bw/day. Treatment was done orally from 30 days. Thereafter body weight, male reproductive organs weight, sperm count, sperm morphology, ACP, ALP, total protein, uric acid and testis and serum testosterone level were determined using standard methods. The changes recorded are indicative of infertility in male rats because of chlorpyrifos exposure. When the subjects were treated with *Emblica officinalis* Garten in conjunction with chlorpyrifos, these parameters exhibited recovery and when treated with *Emblica officinalis* Garten alone, these parameters were more or less near to the control group. This highlights the debilitating effect of chlorpyrifos and scavenging property of *Emblica officinalis* Garten.

**Electronic supplementary material:**

The online version of this article (doi:10.1186/2193-1801-2-541) contains supplementary material, which is available to authorized users.

## Background

Pesticides, a unique group of compounds, are used to prevent, control or eliminate pests which are a major cause of crop losses in the field as well as in storage. The increase in population has resulted in a shift in cultivation of high yielding crops varieties to feed the teeming millions. In conjunction there has been widespread use of pesticides as insecticides, fungicides, herbicides and rodenticides etc. Application of pesticides amplified as the demand for control of pests and their resurgence increased (Giridhar and Indira 1997).

Occupational exposure to pesticides thus became a common and increasingly alarming phenomenon. Around 3 million acute poisonings and 220,000 deaths from pesticide exposure have been reported per annum (Marrs 1993; USDA 1994; Yasmashita et al. 1997). The health effects caused by this occupational exposure are massive and irreversible in some cases. The widespread use of organophosphorus compounds and the high rates of food contamination could leave humans, animals and birds being exposed to high levels of pesticidal toxicity (Suresh Babu et al. 2006).They rapidly spread in the environment, posing potential hazards to human health. These toxic chemicals, which are toxic to target as well as other non-target organisms, become an integral part of the ecosystem. The use of pesticides undoubtedly enhanced during the beginning of the 19th Century.

Chlorpyrifos (CPF) [O,O- diethyl-O (3,5,6 – trichloro-2-pyridyl)]- is one of the pesticides that exhibit a broad spectrum of activity against arthropod and non-arthropod pests of plants, other animals including humans (Breslin et al. 1996). Chlorpyrifos is also known as a residential pesticide for killing fire ants, cockroaches and other household pests. Particularly this pesticide has an effect on behavioral, neurological and reproductive function too (Mueller-Beilschmid 1990). Varying concentrations of chlorpyrifos pollution in the environment has become a common phenomenon (Joshi et al. 2003), posing a potential hazard to human health. It has been reported that chlorpyrifos is linked to human genital deformities.

The chief mechanism of action of OP pesticides occurs by the inhibition of neuronal cholinesterase activity, a key enzyme that is concerned in neurotransmission (Richardson et al. 1993).

*Emblica officinalis* Garten, commonly known as amla (synonym Indian gooseberry), is one of the fruits which contain bioactive components that is thought to have antioxidative properties. As a traditional medicine, widely used in India (Ghosal et al. 1996; Bhattacharya et al. 1999), *Emblica officinalis* Garten enjoys a vital position in Ayurveda, an ancient Indian indigenous system of medicine. It belongs to the family Euphorbiaceae and is distributed in tropical Southeastern Asia, particularly in Central and Southern India (Warrier et al. 1995). For medicinal purpose, fresh or dried fruits are usually used. Unani medicinal system uses dried amla fruits to treat hemorrhage, diarrhea, and dysentery (Parroatta 2001). Apart from being a very rich source of ascorbic acid (Tweari et al. 1982), amla also bears fats, tannins and phyllemblin and minerals like phosphorus, iron, and calcium (Sidhu et al. 2011). *Emblica officinalis* Gaertn leaves and fruit have been used for fever and inflammatory treatments by rural population. The earlier study have demonstrated potent anti-microbial, antioxidant, adaptogenic, hepatoprotective, anti-tumor and anti-ulcerogenic activities in the fruits of *Emblica officinalis*, Leaf extracts have been shown to posses anti-inflammatory activity (Khan 2009).

This study was aimed at to (1) investigate the toxicity of chlorpyrifos on reproductive organs in rats. The body organ weight, sperm morphological abnormality, sperm motility, enzymatic assay, uric acid level and hormonal assay are the criteria used to evaluate the reproductive efficacy of treated rats. (2) see how the herbal product *Emblica officinalis* Garten mitigates the toxicological effects of chlorpyrifos as a bio compatible product.

A large number of compounds have been identified, by different researchers, having protective action against pesticidal toxicity, but those compounds were toxic at their effective dose level*. Emblica officinalis* Garten is found to be a good herbal protector and non-toxic as well, reasonable in cost-benefit aspect and easily available in nature (Chakrawarti et al. 2010).

## Methods

### Animals

Healthy adult male albino rats (*Rattus norvegicus,* Wistar Strain) (weight approx. 170-220 g) were used in the present study. The animals were housed individually in plastic cages, maintaining at a room temperature (21-24°C ± 3°C) in uniform light dark cycle (14:10:L:D).The animals were provided with diet (W.B.Dairy & Poultry Dev. Corp. Ltd.) and water *ad libitum* through out the course of study. Animals were quarantined for 10 days before beginning of the experiments. The work related to rat experimentation was conducted with the permission from ethical committee (Vide ref no 892/ac/05/CPCSEA).

### Chemicals

Chlorpyrifos was obtained from Nagarjuna Agrichem Limited (Hyderabad, Andhra Pradesh, India) for this experiment. All other chemicals were of analytical grade and were obtained from local commercial sources.

*Emblica officinalis* Garten was procured from local market. The fruits were washed, dried and crushed. 20 mg crushed material was extracted with 1 ml of water, and this extracted juice was given to the rat. The dose used as 20 mg *Emblica officinalis* /kg body weight for the experiment for removal of toxicity by *Emblica officinalis* is actually a dose below lethal compared to an oral LD 50 value of 1000 mg/kg body weight in rat. The dose used here is sufficient to carry on their ameliorative properties.

### Animals’ treatment schedule

The oral LD 50 values of any pesticide are not equal and are dependent on the nature of pesticide along with the amount of pesticide exposed to the animals. Accordingly, the oral LD 50 of chlorpyrifos in particular for male rat is 135 mg/kg body weight. The reason for selecting a dose of 12 mg/kg body weight in the present experiment is due to its oral sub lethal dose that caused toxicity to the animals and simultaneously did not cause mortality of the animals. Rats were divided into two groups, control (*n = 5)* and experimental groups *(n = 15)*.The experimental groups were divided into three groups. Group1 (G1) receive 20 mg *Emblica officinalis* Garten/kg bw/d (*n = 5*), group 2 (G2) received 12 mg Chlorpyrifos/kg bw/d *(n = 5*) and group 3 (G3) received 12 mg Chlorpyrifos with *Emblica officinalis* Garten 20 mg/kg bw/d (*n = 5*), through oral intubations. The control groups however received same amount of water. After taking the body weight, both control and experimental rats were sacrificed after 30 days of treatment and samples were taken for organ weight measurement, sperm motility analysis, sperm density, testicular sperm count, epididymal sperm morphology, quantitative study of protein, measurement of uric acid level, estimation of testosterone level in blood and serum, acid phosphatase and alkaline phosphatase activity.

### Body and organ weights

The body weight has been recorded on the initial day of experiment and also on the day of sacrifice (31^st^ day), both the control and experimental groups, by using automatic balance. The increment of body weight is presented in percentage. Similarly weight of different reproductive organs (Testis, Seminal vesicle and Epididymis) was also recorded.

### Testicular sperm count

Immediately after dissection, one testis of each rat was placed in 1 ml phosphates buffer (pH 7.4).Tunica albuginea was cut by surgical blades, removed and the remaining semeniferous tubules were mechanically minced using surgical blades in 1 ml phosphate buffer. The testicular cell suspension was pipetted several times to make a homogenous cell suspension. One drop of the suspension was placed on the “Hemocytometer chamber” (Neubauer improved, Feinoptik Bad Blankenburg, Germany) and testicular sperm suspension was evaluated as million sperm cells per ml of suspension under 200X magnification using phase contrast microscope and the sperm were Counted manually. Testicular sperm count was measured by Uzunhisarcikli et al. (2007).

### Sperm motility analysis

Sperm were collected as quickly as possible after a rat was dissected. The epididymis was cut by surgical blades into 1 mm^3^ pieces approximately in 1 ml phosphate buffer saline solution at 37°C. The solution was pipetted several times in order to homogenize the sperm suspension and one drop of the suspension was placed on a slide, covered by 24 × 24 mm cover slips and evaluated under 100X × 10X magnification using phase contrast microscope. Sperm motility was categorized in to “motile” or “immotile”. Results were recorded as percentage of sperm motility (Uzunhisarcikli et al. 2007).

### Epididymal sperm morphology

Sperm morphology was assessed by the method of Filler (1993). The epididymis was removed and placed in 2 ml of 0.9% saline. It was minced and allowed to incubate for 15 min at 37°C. The sperm were evaluated microscopically at 40X × 10X magnification for identifying the head, tail, mid piece and other sperm abnormalities. At least 300 sperm were evaluated from each slide.

### Sperm density

The reproductive organ epididymis was removed and fixed in Bouins fixative for 12–14 hrs. It was processed in a series of graded ethanol and embedded in paraffin. Section were cut at 5 μm thickness and stained with hematoxylin and eosin for light microscopic examination (10X × 10X). The qualitative changes were recorded. In the lumen of the epididymis sperm density was observed and was graded as normal (+++), moderately decreased (++), or severely decreased (+), depending on the concentration of spermatozoa in the tubular cross-sections through the microscope.

### Quantitative study of protein (Testis, Seminal vesicle, Epididymis)

Total quantity of protein was estimated by the method of Lowry et al. (1951).

### Estimation of uric acid

Uric acids level was quantified by Serum Uric acid Kit (1972).

### Estimation of testosterone in testis and serum

Testosterone in testis and serum level was estimated by method of Mukherjee et al. (2006) and Orcyzk et al. (1979).

### Estimation of acid phosphatase

Acid phosphatase enzyme activity was determined by the method of Walter and Schutt (1974). The amount of 4-Nitrophenol in the medium was estimated by measuring the yellow colour at 405 nm in a UV-visible Spectro photometer (Varian,Cary 50 Bio) against an analysis blank.

### Estimation of alkaline phosphatase

Activity of alkaline phosphatase was estimated using the method of Ohmori (1937) with slight modification. The reaction product was measured at 405 nm in a UV-visible Spectro photometer (Varian,Cary 50 Bio) against the analysis blank. Activity was determined in the same way as done in estimation of acid phosphatase.

### Statistical analysis

Data were statistically analyzed using *t*-test. The maximum significant level chosen was *P < 0.05*.

## Results

### Evaluation of body weights

Death was not observed in any of the experimental groups during experimental periods. But especially food intake in group 2 reduced during the experiment. It is observed from the Table 1, that the body weight has increased with the advancement of age, both in control and in the treated groups. The body weight was noted to be decreased significantly (*P <* 0.001) for 30 days treatment of chlorpyrifos treated group (G2) and when *Emblica officinalis* Garten was fed singly for 30 days, the body weight increased significantly as compared to control. But when *Emblica officinalis* Garten was fed with chlorpyrifos (G3), the body weight regained significantly (*P < 0.001*) than the chlorpyrifos treated group (G2), suggesting some remedial role of amla.

**Table 1:** **Body weight after 30 days treatment**

Day & dose	No of animal exposed	Initial body weight (gm)	Final body weight (gm)	Body weight increased (%)
**Control**	**5**	180.40	212.49	17.78
		± 4.54	± 5.40	± 0.09
**G1**	**5**	188.42	224.00	19.51***
		± 2.94	± 2.91	± 0.20
**G2**	**5**	186.60	201.70	8.07***
		± 3.78	± 4.40	± 0.31
**G3**	**5**	190.50	217.00	13.95***aaa
		± 3.45	± 3.87	± 0.16

All data are presented as mean ± S.E.M from five similar experiments. Data are significant at the level ***P < 0.001, for G1 G2 and G3 than the control animals. aaa P < 0.001, for G3 than the G2.

### Evaluation of organ weight

The absolute and relative weights of testis, seminal vesicle and epididymis were found to be decreased significantly (*P < 0.001*) in CPF treated group (G2) than the control group (Table 2). When *Emblica officinalis* was singly fed, the absolute and relative weights of experimental organs showed no significant changes than the control. Due to recovery effects, when *Emblica officinalis* was fed with chlorpyrifos in G3 group, the reproductive organ weight has significantly increased (*P < 0.001, P < 0.05*, *P < 0.01*), in testis, seminal vesicle and epididymis respectively, than the chlorpyrifos treated rat, though having a significant difference than the control one.

**Table 2:** **Organ weight after 30 days treatment**

Day & dose	No of animal exposed	Testis (gm)		Seminal vesicle (gm)		Epididymis (gm)	
		Absolute weight of body weight	Relative weight of body weight	Absolute weight of body weight	Relative weight of body weight	Absolute weight of body weight	Relative weight of body weight
**Control**	**5**	1.88	0.89	0.75	0.35	0.8	0.38
		± 0.06	± 0.03	± 0.04	± 0.01	± 0.03	± 0.01
**G1**	**5**	2.03	0.90	0.81	0.36	0.85	0.38
		± 0.05	± 0.01	± 0.04	± 0.02	± 0.03	± 0.01
**G2**	**5**	0.97***	0.48***	0.39***	0.19**	0.42***	0.21***
		± 0.05	± 0.02	± 0.03	± 0.02	± 0.04	± 0.02
**G3**	**5**	1.53**aaa	0.71***aaa	0.59*a	0.27*a	0.68aa*	0.32**aa
		± 0.02	± 0.01	± 0.04	± 0.02	± 0.03	± 0.01

All data are presented as mean ± S.E.M from five similar experiments. Data are significant at the level ***P < 0.001, **P < 0.01, and *P < 0.05 for G1, G2 and G3 than the control animals. aaa P < 0.001, aa P < 0.01 and a P < 0.05 for G3 than the G2.

### Evaluation of testicular sperm count

**Figure 1:**
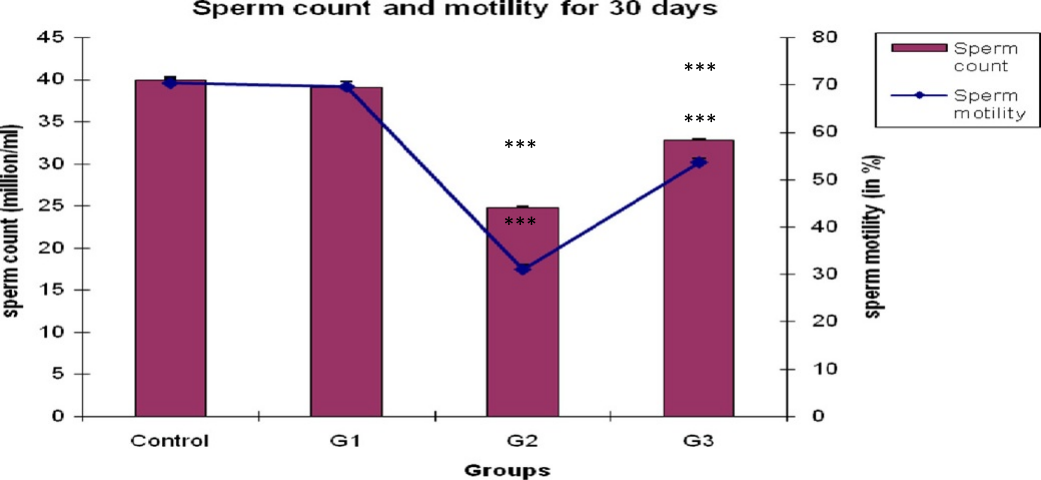
**Bar and graphical representation of sperm count and sperm motility after 30 days exposure.** ***P <0.001 for G2 and G3 than the control animals.

It is observed from Figure 1 that the sperm count of CPF treated group, G2, significantly decreased (*P <* 0.001) then the control group. However, when *Emblica officinalis* was fed with CPF for 30 days, the sperm count significantly increased (*P <* 0.001) than the CPF treated group G2, but still has a significant difference than the control rats. Singly fed *Emblica officinalis* have no significant change in testicular sperm count as compared to the control rats.

### Evaluation of epididymal sperm morphology

A significant decline (*P <* 0.001) was found in normal sperm morphology in CPF treated group, G2, than the control group. Simultaneously, a significant increment (*P <* 0.001) was recorded in abnormal sperm morphological structures in CPF treated group for 30 days of exposure (Table 3). Thick coil tail, tapered head and without head sperm morphologies are the criteria of abnormal sperm morphology. In recovery aspect, when CPF was administered with *Emblica officinalis* (G3), the sperm morphological deformities (thick coil tail, tapered head and without head) significantly decreased (*P <* 0.001) than the CPF treated group G3, still having a significant difference (*P <* 0.001) than the control group. No statistically significant changes were detected in normal sperm as well as abnormal sperm morphology in sole *Emblica officinalis* treatment in the group (G1) than the control group.

**Table 3:** **Sperm morphology (%) after 30 days treatment**

Day & dose	Normal morphology	Abnormal morphology	Thick coil tail	Tapared head	Without head
**Control**	94.968	5.032	3.772	4.488	3.89
	± 0.211	± 0.211	± 0.061	± 0.105	± 0.103
**G1**	94.526	5.474	3.932	4.936*	3.934
	± 0.107	± 0.107	± 0.124	± 0.116	± 0.068
**G2**	66.856***	33.144***	11.838***	13.728***	16.850***
	± 0.237	± 0.237	± 0.136	± 0.066	± 0.158
**G3**	86.132 aaa	13.868 aaa	7.464 aaa	7.332 aaa	8.978 aaa
	± 0.105***	± 0.105***	± 0.118***	± 0.058***	± 0.114***

All data are presented as mean ± S.E.M from five similar experiments. Data are significant at the level ***P < 0.001 and *P < 0.05 for G1, G2 and G3 than the control animals. aaa P < 0.001 for G3 than the G2.

### Evaluation of sperm motility analysis

A significant decline (*P <* 0.001) was observed in total epididymal sperm motility at the end of 30^th^ day in CPF treated rat than the control group (Figure 1). But when *Emblica officinalis* was fed with CPF, the sperm motility increased significantly (*P <* 0.001) than the CPF treated group G2. However, it still has significant difference (*P <* 0.001) than the control rat’s sperm motility. Single *Emblica officinalis* fed group have no significant difference than the control group.

### Sperm density

**Figure 2:**
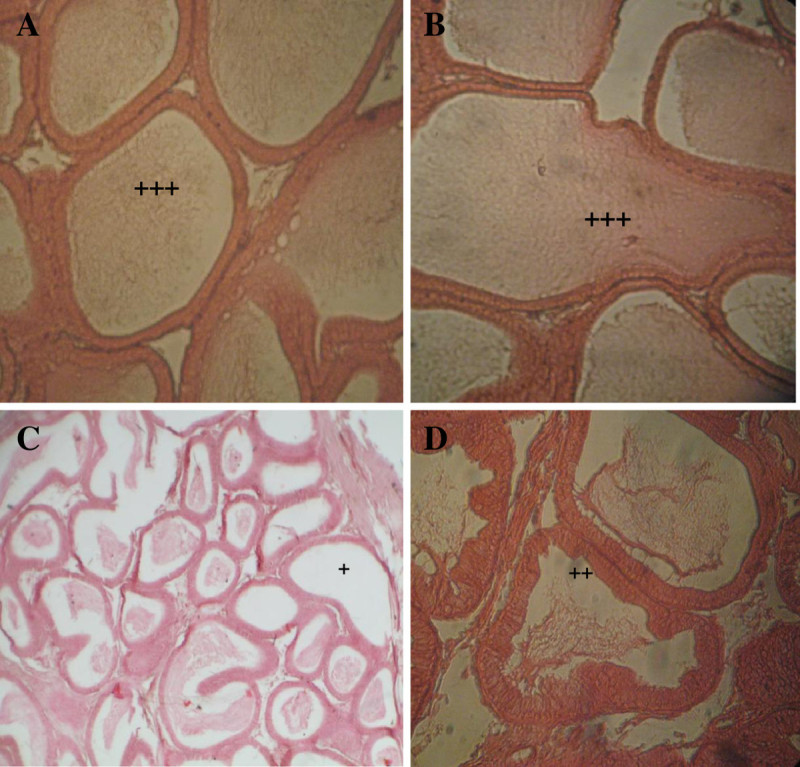
**T.S. of epididymis in control rat (A) showing normal sperm density (+++) in epididymal lumen, in** ***E*** **.** ***officinalis*** **treated rat (B) showing normal sperm density (+++) in epididymal lumen, in chlorpyrifos treated rat (C) showing severely decreased (+) and in chlorpyrifos with** ***E*** **.** ***officinalis*** **treated rat (D) showing moderate decline(++) sperm density in epididymal lumen.**

Due to CPF treatment, sperm density was reducing in the lumen of epididymal tubule in respected to control rats (Table 4). In epididymis, the control animals showed normal sperm density (+++). However, the sperm density severely decreased (+) in CPF treated group G2. However, when CPF was fed with *Emblica officinalis*, the sperm density shows a moderate decline (++). Single *Emblica officinalis* fed group showed normal sperm density (Figure 2).

**Table 4:** **Sperm density (microscopically observed) for 30 days treatment**

Group	Sperm density
Control	+++
G1	+++
G2	+
G3	+++

+++ showing normal sperm density, ++ showing moderately decreased,+ showing severely decreased sperm density.

### Quantitative estimation of protein

According to Table 5, protein level of testis, epididymis and seminal vesicle significantly increased *P <* 0.001 in CPF treated group G2, than the control group. However, when animals were simultaneously treated with CPF and *Emblica officinalis*, the protein level significantly decreased than the G2 group, although these values have a significant difference than the control groups in testis, epididymis and seminal vesicle respectively. When *Emblica officinalis* was singly fed for 30 days, the reproductive organs except epididymis (*P <* 0.001) have no significant changes than the control value.

**Table 5:** **Protein estimation (mg/g) of testis, epididymis and seminal vesicle after 30 days treatment**

Organ	Testis	Epididymis	Seminal vesicle
**Control**	8.008	4.822	7.738
	±0.019	±0.024	±0.047
**G1**	8.054	5.548***	7.842
	±0.015	±0.030	±0.013
**G2**	12.772***	10.330***	11.644***
	±0.039	±0.021	±0.013
**G3**	8.834***	7.030***	9.484***
	±0.037 aaa	±0.027 aaa	±0.027 aaa

All data are presented as mean ± S.E.M from five similar experiments. Data are significant at the level ***P < 0.001 for G1, G2 and G3 than the control animals. aaa P < 0.001 for G3 than the G2.

### Estimation of Uric acid

**Figure 3:**
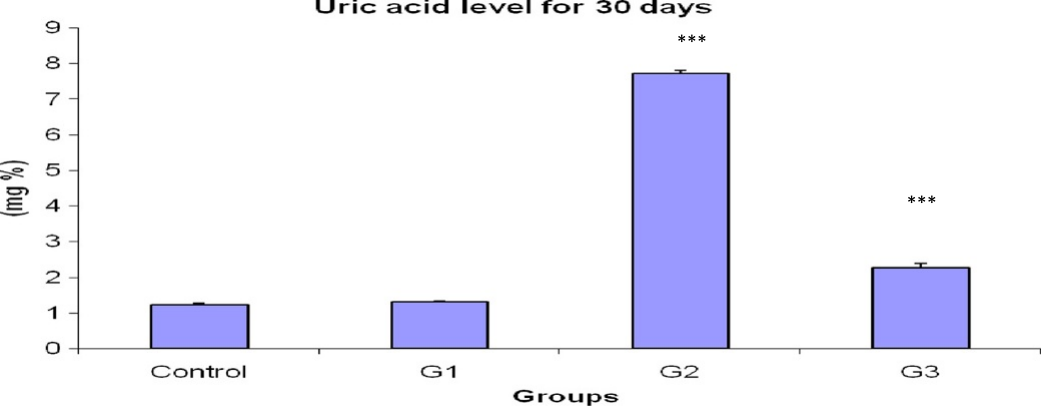
**Bar diagram of uric acid level after 30 days exposure.** ***P < 0.001 for G2 and G3 than the control animals.

A significant rise (*P <* 0.001) was found in uric acid level (Figure 3) of G2 group as compared to the control value. When CPF was fed with *Emblica officinalis*, the uric acid level significantly decreased (*P <* 0.001) than the CPF treated G2 group, but is still significantly higher than the control group. However, when *Emblica officinalis* was fed for 30 days singly, the uric acid level has no significant difference than the control value.

### Estimation of testosterone in testis and serum

**Figure 4:**
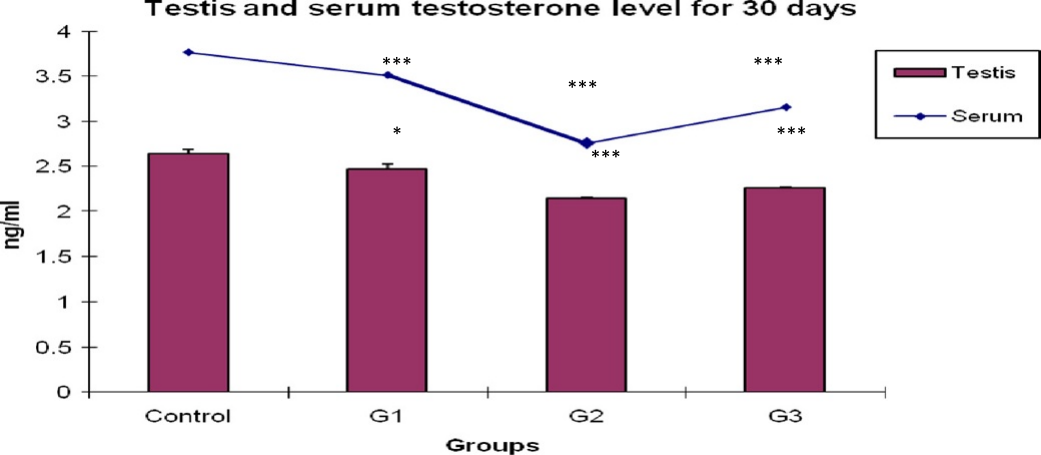
**Bar and graphical representation of testis and serum testosterone level after 30 days exposure.** ***P < 0.001,*P < 0.05 for G1, G2 and G3 than the control animals.

As shown in Figure 4, the testosterone level of testis and serum was significantly lowered (*P <* 0.001) in CPF treated G2 group as compared to the control value. In recovery aspect, when *Emblica officinalis* was fed with CPF for 30 days, the testosterone level of testis and serum were observed to be significantly higher (*P <* 0.001) than the CPF treated group G2, but still is significantly lower than the control value. When *Emblica officinalis* was singly fed for 30 days, the testosterone level of testis and serum showed a significant decline (*P <* 0.05 and *P <* 0.001 respectively) than the control value.

### Estimation of acid phosphatase (ACP)

The acid phosphatase level of testis, epididymis and seminal vesicle were significantly decreasing *P <* 0.001 in CPF treated group G2 than the control value. However in G3, where *Emblica officinalis* was fed with CPF, the acid phosphatase level showed a significant rise *P <* 0.001 in respective tissues than the CPF treated group G2, but this value showed a significant difference *P <* 0.01 than the control value. Singly fed *Emblica officinalis* group showed no significant change in acid phosphatase level of testis, epididymis and seminal vesicle than the control value (Table 6).

**Table 6:** **Acid phosphatase activity (ACP) (μ p-nitrophenol/ mg tissue) of testis, epididymis and seminal vesicle after 30 days treatment**

Organ	Testis	Epididymis	Seminal vesicle
**control**	3.666	3.766	2.774
	±0.009	±0.027	±0.016
**Amla 20 mg G1**	3.626	3.728	2.738
	±0.018	±0.018	±0.007
**12 mg CPF G2**	1.974***	2.330***	0.878***
	±0.010	±0.021	±0.011
**12 mg CPF + 20 mg Amla G3**	3.562**	3.638**	2.680**
	±0.022 aaa	±0.012 aaa	±0.015 aaa

All data are presented as mean ± S.E.M from five similar experiments. Data are significant at the level ***P < 0.001,**P < 0.01 for G1, G2 and G3 than the control animals. G2, aaa P < 0.001 for G3 than the G2.

### Estimation of alkaline phosphatase (ALP)

The alkaline phosphatase level of testis and seminal vesicle were significantly lower and that of epididymis was significantly higher (*P <* 0.001) than the control value. However in *Emblica officinalis* treatment, G3 group shows a significant increment in alkaline phosphatase level *P <* 0.001 in testes and seminal vesicle and significant decline in epididymis than the G2 group; though these values still have a significant difference than the control group (Table 7).

**Table 7:** **Alkaline phosphatase (ALP) (μ p-nitrophenol/ mg tissue) activity of testis, epididymis and seminal vesicle after 30 days treatment**

Organ	Testis	Seminal vesicle	Epididymis
**Control**	8.186	7.838	1.400
	±0.010	±0.012	±0.024
**G1**	8.168	7.820	1.432
	±0.012	±0.009	±0.009
**G2**	4.802***	4.768***	6.386***
	±0.018	±0.022	±0.014
**G3**	7.052***	6.298***	1.688***
	±0.020 aaa	±0.017 aaa	±0.021 aaa

All data are presented as mean ± S.E.M from five similar experiments. Data are significant at the level ***P < 0.001 for G1, G2 and G3 than the control animals. G2, aaa P < 0.001 for G3 than the G2.

## Discussion

The exponential increase in the production and extensive use of pesticides has a profound impact on the environment and creates unforeseen hazards to any organism as well as man (Chia 2000; Karallieda et al. 2003; Karanthi et al. 2004). Organophosphates are among the most widely used synthetic pesticides. The wide spread use of organophosphate insecticides (OPIs) has a causable toxic effect on reproductive system (Joshi et al. 2007).

Reproductive organ weights are the criteria used for evaluation of reproductive toxicity (Zidan 2009). In general toxicity studies, it is well known that the alterations in body and organ weights are sensitive indicators of the detection of potentially toxic chemicals. In our study, during the exposure toxic symptoms were observed. The body and organ weight were decreased during chlorpyrifos exposure. It is agreed that the weight of reproductive organs decreases significantly at various dose levels (7.5, 12.5 and 17.5 mg/kg bw/day) of CPF for 30 days of treatment (Joshi et al. 2007). Decrease in testis weight could be a most sensitive parameter indicating the male gonadal toxicity. Similar results were found by Chitra et al. (1999), where body weight and testicular weight were reported to be decreased significantly in endosulfan treated rats, indicating impairment at testicular functions affecting androgenesis. Testicular steroidogenesis is regulated by hypothalamo-pitutary axis, which might be distressed by toxic inputs (Singh and Pandey 1989). The epididymis and seminal vesicles both are androgen-dependent organs. Testosterone is more essential for their growth and function and a reduction in their weights may reflect a decline in bioavailability and production of androgens. A similar type of decrease was found in body weight and reproductive organs weight of adult male rat for 90 days treatment of pirimiphos-methyl exposure (Ngoula et al. 2007). Weight of testis and epididymis were significantly lowered in the profenofos treated rats. The decrease in testicular weight in treated rats may be due to reduction of tubule size, spermatogenic arrest and inhibition of steroid biosynthesis of Leydig cells (Sujatatha et al. 2001; Kaur and Mangat 1980). Similar results were recorded by Chadhuary and Joshi (2003), who reported a significant reduction in the rat testis weight after exposure of endosulfan for 15 and 30 days, at the dose levels of 5, 10 and 15 mg/kg bw/day. In addition, EL-Kashoury (2009), showed that the weight of testis was significantly lowered in male rats exposed to profenofos at the dose of 23.14 mg/kg bw for 60 days treatment. According to Zidan (2009), the reproductive organ weights (testis, epididymis and seminal vesicle) of male rats were significantly lowered at the dose level of 5 and 50 ppm of chlorpyrifos-methyl, diazinon and profenofos treatment for 65 days. The weight of testis and accessory sex organs are known to be dependable indicator of testicular androgen production (Price and Willams-Ashman 1961; Rind et al. 1963). Significant decrease in testicular weight may be a cause of decrease in the number of spermatogenic elements and spermatozoa (Sherins and Hawards 1978; Takihara et al. 1987). Abd El-Aziz et al. (1994) found that diazinon treatment decreased the weights of most genital organs when administered at two different doses of 1.5 and 3 mg/kg body weight in male rats for 65 consecutive days. The reduction of organ weights may be due to pesticides exposure which is affecting their hypothalamus, pituitary or both Okazaki et al. (2001). In the present study it is found that when amla was fed with CPF, there was a significant recovery seen in body and organ weight than the Chlorpyrifos treated group. It may be due to recovery of organ or tissue injury or due to revitalization of androgen secretion. Similarly, when amla singly fed, the body weight was increased, as compared to the control group. Its may be some adverse effects of different constituents of aqueous extract of *Emblica officinalis*. According to Mode et al. (2009), broiler birds gain their body weight due to amla treatment for 28 to 42 days. This body weight gain might be due to the hepatoprotecting activity resulting in the improvement in the liver function (Pande and Zeestress 2000; Babu et al. 2002; Ratankumar et al. 2004). According to Singh et al. (2006), *Emblica officinalis* showed a recovery effect in body weight until 30 days of post-irradiation treatment.

A parallel essential indicator is sperm cell degeneration. The sperm count is one of the most sensitive tests for spermatogenesis and it is highly correlated with fertility. According to our study the sperm morphological abnormalities were increased, sperm count, sperm motility and density were decreased due to Chlorpyrifos treatment. Similar type of result was found in 5.4 and 12.8 mg/kg/d Chlorpyrifos treated rat group during 90 days treatment. The decrease of sperm motility and density after oral treatment of chlorpyrifos may be due to inadequacy of androgen (Chadhuary and Joshi 2003), which caused anorgasmia in testicular functions by altering the activities of the enzymes which is causative for spermatogenesis (Siha et al. 1995; Reuber 1981). Similar type of results was found in 23.14 mg/kg body weight profenofos treated rats for 60 days (EL-Kashoury 2009). According to Zidan (2009), percentage of sperm motility and sperm count significantly decreased in both the three pesticides (chlorpyrifos methyl,dizinon and profenofos). Simultaneously total sperm abnormalities significantly increased for all the tested pesticides. According to our result thick coil tail, tapered head and without head were the selected parameters for sperm abnormalities studies, without head abnormalities showing maximum percentages, and these anomalies are considered as a better discriminator between fertile and infertile males (Guzik et al. 2001). Sperm morphology and motility are useful markers of toxic damage even in absence of any effect on male fertility. Two main regulatory processes, endocrine regulation via the gonadotropin hormones and local regulation via inter-cellular communication, control spermatogenesis. (Holdcraft and Braun 2004). The similar result was showed by Abd El-Aziz et al. (1994)*,* who revealed that diazinion treated rats show decreased sperm motility associated with an increment of dead sperm percentage. Prior epidemiologic work on Chinese pesticide factory workers showed that OP exposure was associated with decreased sperm concentration and motility (Padungtod et al. 2000). It is established that, sperm motility is an important functional measurement to anticipate sperm fertilizing capacity (Aikten et al. 1984). Any negative impact on motility would seriously affect fertilizing ability of the organism (Murugavel et al. 1989). Low level of ATP content seriously affects the sperm motility. Sperm motility may be affected by alteration of the enzymatic activities of oxidative phosphorolytic process (Tso and Lee 1981). Similarly oxidative phosphorolytic process is required for ATP production; it is a source of energy for the alleviated movement of spermatozoa (Joshi et al. 2007). Full ATP pool is crucial for normal spermatozoal movement and a slight deprivation of ATP leads to reduction in motility, which is one of the major causes of infertility (Poon et al. 2004). The decrease of sperm density in the epididymis is one of the indicators of reduction in spermatogenesis owing to the toxicity of any agent (Poon et al. 2004). Decline in sperm density may be due to direct spermicidal effects presence on Chlorpyrifos treated rats epididymis. The obtained results are in accordance with Narayana et al. (2006), who revealed that the sperm density of adult male rats was decreased due to various dose of methyl parathion exposure.

According to Chakraborty and Rm (2009), oral administration of aqueous extract of *Emblica officinalis* along with ochratoxin for 45 days significantly mitigates ochratoxin-induced alterations in reproductive parameters. The recovery aspect of the herbal product, *Emblica officinalis*, find similar light in our study, where there was a significant increase in sperm count, normal sperm morphology and sperm motility. This shows the ameliorative effect of *Emblica officinalis*, which might be due to the presence of bioactive compounds, namely: emblicanin A, emblicanin B, punigluconin and pedunculagin which are known to provide protection against free oxygen radicals in various *in vitro* studies (Bhattacharya et al. 1999).

Chlorpyrifos also induces biochemical changes in testis, epididymis and seminal vesicle. Our result reveals that the protein content was significantly elevated in male reproductive organs due to chlorpyrifos exposure. According to Joshi et al. (2007), the protein content of testis was significantly increased in chlorpyrifos treatment during 30 days exposure. Similar results showed the same trend in the protein content caused by several pesticides, at different exposure levels and or different concentrations, as reported by El-Kashoury and Tag El-Din (2010); EL-Kashoury (2009); Shivanandappa and Krishna Kumar (1981); Bulusu and Chakravarty (1992); Joshi et al. (2003) and Ngoula et al. (2007). Puga et al. (1974) demonstrated that the elevation of protein content may be due to the stimulation of growth proteins and RNA synthesis. Dikshith and Dutta (1972), Gupta et al. (1981) and Singh and Pandey (1989) showed that an elevation in the testicular protein may be due to the hepatic detoxification activities which resulted in the inhibitory effect on the activities of enzymes involved in the androgen biotransformation. In accordance with the findings of the present study, Rao and Chinoy (1983), suggested that the accumulation of protein occurred in testis epididymis due to androgen deprivation to target organs and this deprivation effect also led to a reduction in testicular and cauda epididymal sperm population, loss of motility in the latter and an increase in the number of abnormal spermatozoa. Chakrawarti et al. (2010) reported an earlier and faster recovery in *Emblica officinalis* treated groups. They reveal that the total protein content was adversely affected by cadmium with *Emblica officinalis* showing a protective action of the latter against cadmium treated group. A significant increase in the number of ribosome may be occurring due to their increased mobilization from ER and this leads to the augmented protein synthesis (Mukerjee and Goldfeder 1974).

Our result reveals that due to chlorpyrifos exposure uric acid level was significantly increased in serum. Similar result was found in various doses of methyl parathion treated rats (Prashanthi et al. 2006; Narayana et al. 2006). This may be due to stress induced toxicity leading to increased uric acid level in blood serum. The changes were less severe in *Emblica officinalis* treated group suggesting a protection against pesticides. *Emblica officinalis* is one of the richest sources of vitamin C and it mitigates the uric acid level in blood serum.

A significant reduction in ACP and ALP level was found in testes tissue of chlorpyrifos treated rats in the present study, reflecting suppression in testicular function (Johnson et al. 1970) and indicating a nonfunctional spermatogenesis. Our result is supported by the finding of Prashanthi et al. (2006) and Narayana et al. (2006). They revealed that the ACP level was significantly decreased in methyl parathion induced rat’s epididymis. According to EL-Kashoury (2009), the ACP and ALP level was significantly decreased in profenofos treated testicular tissue of male rats. Chlorpyrifos induced cell damage results in the release of ACP and ALP into the blood stream, hence reducing its level in the reproductive tissue. This is similar to the findings of Abraham and Wilfred (2000). Decline in ALP activity indicated that chlorpyrifos treatment created a state of decreased steroidogenesis where the intra- and inter-cellular transports were reduced as the metabolic reactions channelize the required inputs for steroidogenesis (Yousef et al. 2001). Acid phosphatase is enzyme competent of hydrolyzing orthophosphoric acid esters in an acid medium. The testicular acid phosphatase gene is up regulated by androgens and is down-regulated by estrogens (Yousef et al. 2001), when the androgen production is inferior, may be the ACP activity is sermonized.

Due to chlorpyrifos exposure reduction in the serum testosterone level is demonstrated by Joshi et al. (2007). Our result is focusing the same light. Similar observation was noted by Zidan (2009), who revealed that there is significant alteration in chlorpyrifos methyl, diazinon and profenofos treated male rat testosterone. He also stated that testosterone is the principal male hormone produced by the interstitial Leydig cells of testes. Thus testes are responsible for the synthesis of the male sex hormones; so the decrease in testosterone level might be due to an extensive damage of Leydig cells. Besides, disorders of male genital function (hypogonadism) are manifested by a decrease in plasma testosterone level. Hypogonadism may occur with faulty seminiferous tubular function or defective Leyding cell function and this leads to aridity through decreased production of spermatozoa (Zidan 2009).

Biochemical and hormonal estimations of various parameters indicated that the values of *Emblica officinalis* treated groups were near the control values. *Emblica officinalis* extract has been shown to have antioxidant and antiperoxidant properties due to the presence of tanoids, mainly emblicanin-A, emblicanin-B, punigluconin, pedunculogin gallic acid (Bhattacharya et al. 1999)and also steroid (Gupta et al. 2013). The *in vitro* antioxidant activity of tannoids was demonstrated by Ghosal et al. (1996). Some of the plants like *Glycyrrhiza Glabra* (liqurice), *Rubia Cordifolia* (Family-Rubiaceae) and *Phyllanthus*, *Emblica* have also been reported to possess antioxidant and free radical scavenging activities (Jose and Kuttan 1995; Tripathi et al. 1997; Korina and Afanasav 1997). The emblicanin are probable to the major antioxidant principles and it also reported that the antioxidant action present *in vitro* (Ghosal et al. 1996) and *in vivo* (Bhattacharya et al. 1999; Bhattacharya et al. 2000). *Emblica officinalis* is the rich sources of vitamin C, minerals and amino acids and also contains a wide variety of phenolic compounds (Rajkumar et al. 2011), those are the excellent scavengers of oxygen free radicals within the cells where reactive metabolites are produced (Uzunhisarcikli et al. 2007) by chlorpyrifos toxicity. Steroids are present in water soluble *Emblica officinalis* extract (Gupta et al. 2013), that may mimic the normal function of testosterone which plays an important role in reproductive development in mammals. But any of the other constituents of aqueous extract of *Emblica officinalis* may inhibit the secretion of testosterones when administrated singly amla*.*

**Figure 5:**
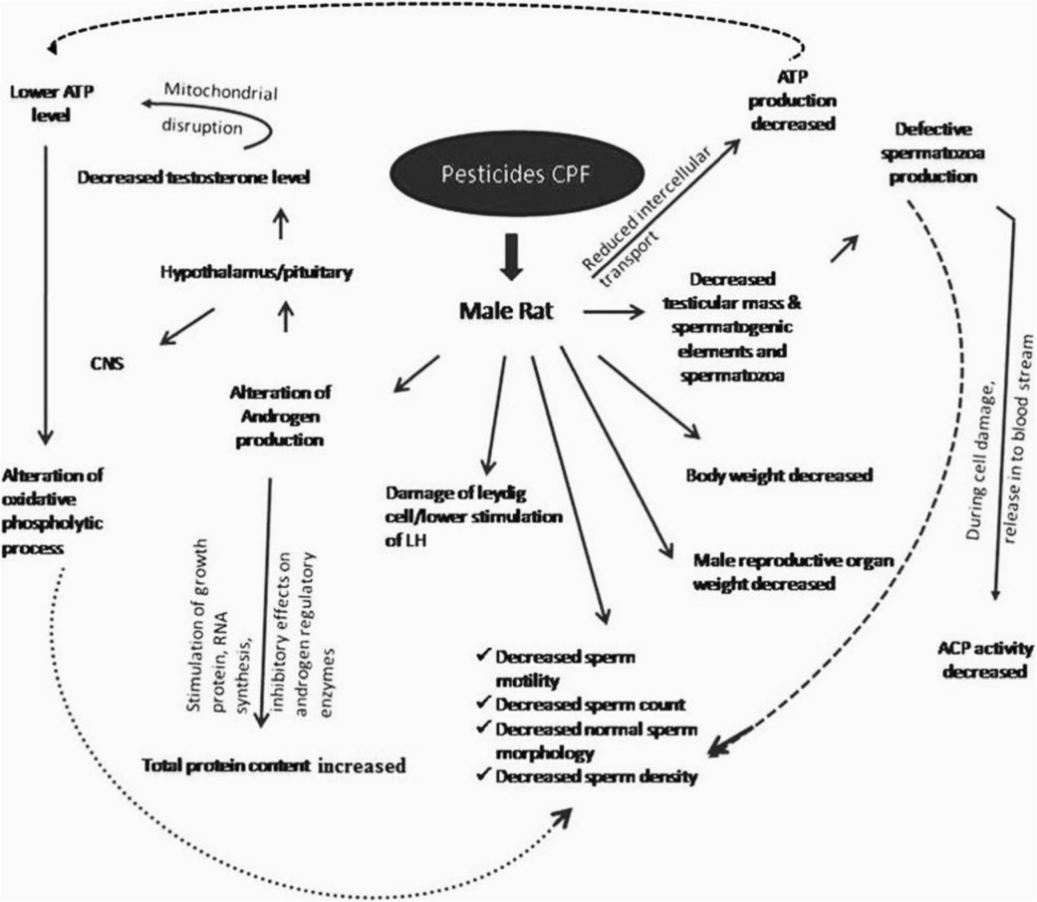
**Schematic diagram showing the effects of pesticides on testicular tissue and the probable pathways of damage.**

**Figure 6:**
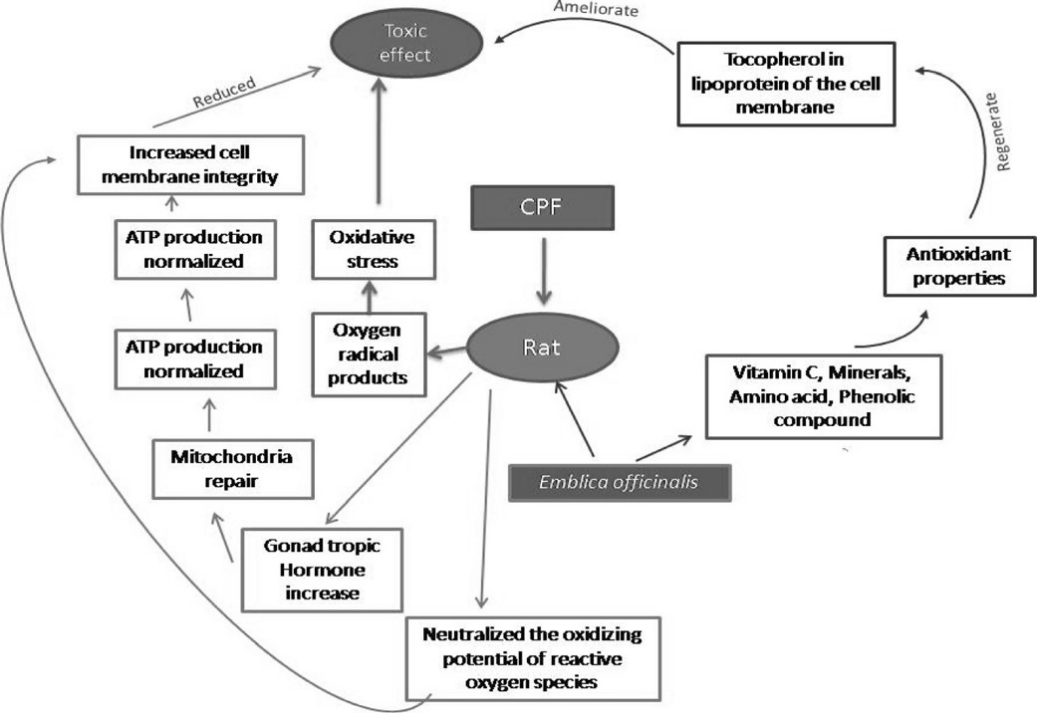
**Diagram showing the probable mechanism of remedial effects of** ***Emblica officinalis*** **against the pesticides treated tissue.**

The stress pathways caused by CPF and ameliorating pathways by which *Emblica* acts are presented schematically in (Figure 5 and 6).

## Conclusion

Under the light of this study, it is concluded that chlorpyrifos is responsible for irreversible damage to male reproductive organs as well as decreased in body weight, sperm morphology, sperm count, sperm motility and sperm density, ACP, ALP and testosterone level, simultaneously significantly increase in abnormal sperm morphology, protein and uric acid level. These changes are potentially harmful and lead to reproductive infertility in rats. Our results reveal that chlorpyrifos induced oxidative stress diminishes the male fertility, thus being harmful to any animal, especially mammals like the human being.

Based on the results obtained it can be concluded that aqueous extract of *Emblica officinalis* Garten formulation, an herbal preparation ameliorate male reproductive tissue damages. Aqueous extract of *Emblica officinalis* Garten contains antioxidants, several flavonoids (Khan 2009) and steroids, these reduces the oxidative stress and recover the testicular tissue damage. *Emblica officinalis* fruit juice neutralizes the oxidizing potentials of reactive oxygen species induced by chlorpyrifos; through, these activities they maintain cell membrane integrity and viability. The present study mainly indicates that *Emblica officinalis* Garten play a core role to reduce the chlorpyrifos toxicity in male reproductive aspect. Any of the following ingredients may exert an inhibiting effect on testosterone secretion, that’s why testosterones level is decreased. Details study of different constituents of aqueous extract of *Emblica officinalis* separately feed is needed to find out the possible reasons of decreased testosterone level in serum and testis.

## Authors’ original submitted files for images

Below are the links to the authors’ original submitted files for images. Authors’ original file for figure 1 Authors’ original file for figure 2 Authors’ original file for figure 3 Authors’ original file for figure 4 Authors’ original file for figure 5 Authors’ original file for figure 6 Authors’ original file for figure 7 Authors’ original file for figure 8
