# Supplementary figures and images for: Emblica officinalis Garten fruits extract ameliorates reproductive injury and oxidative testicular toxicity induced by chlorpyrifos in male rats
Source: Springerplus. 2013 Oct 17;2(1):541. doi: 10.1186/2193-1801-2-541 (PMC3824715; doi:10.1186/2193-1801-2-541)

**Sperm count and motility for 30 days**

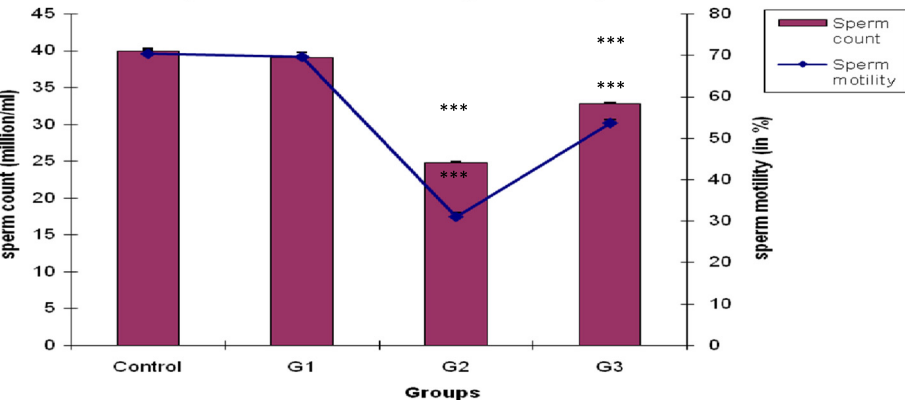

Supplement: Supplementary file 1 — Authors’ original file for figure 1 [file 40064_2013_598_MOESM1_ESM.pdf]

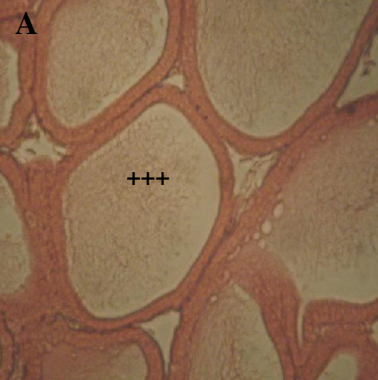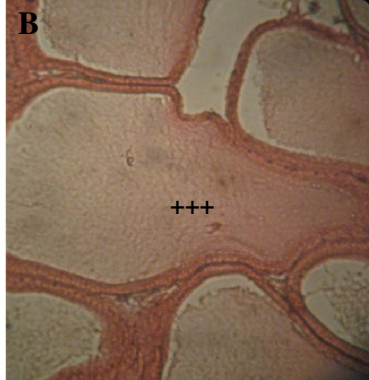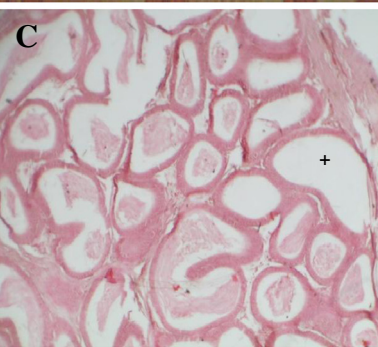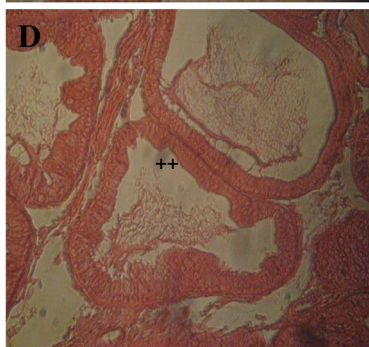

Supplement: Supplementary file 2 — Authors’ original file for figure 2 [file 40064_2013_598_MOESM2_ESM.pdf]

Uric acid level for 30 days

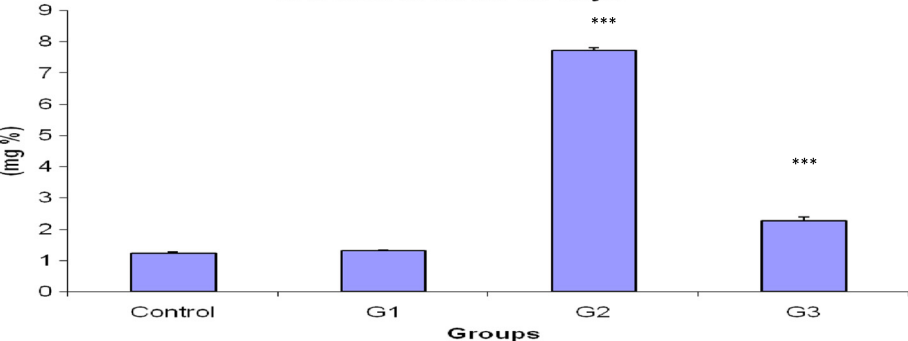

Supplement: Supplementary file 3 — Authors’ original file for figure 3 [file 40064_2013_598_MOESM3_ESM.pdf]

Testis and serum testosterone level for 30 days

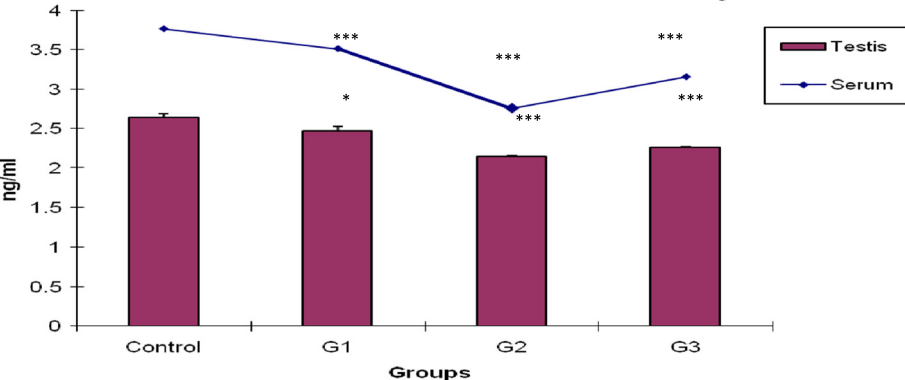

Supplement: Supplementary file 4 — Authors’ original file for figure 4 [file 40064_2013_598_MOESM4_ESM.pdf]

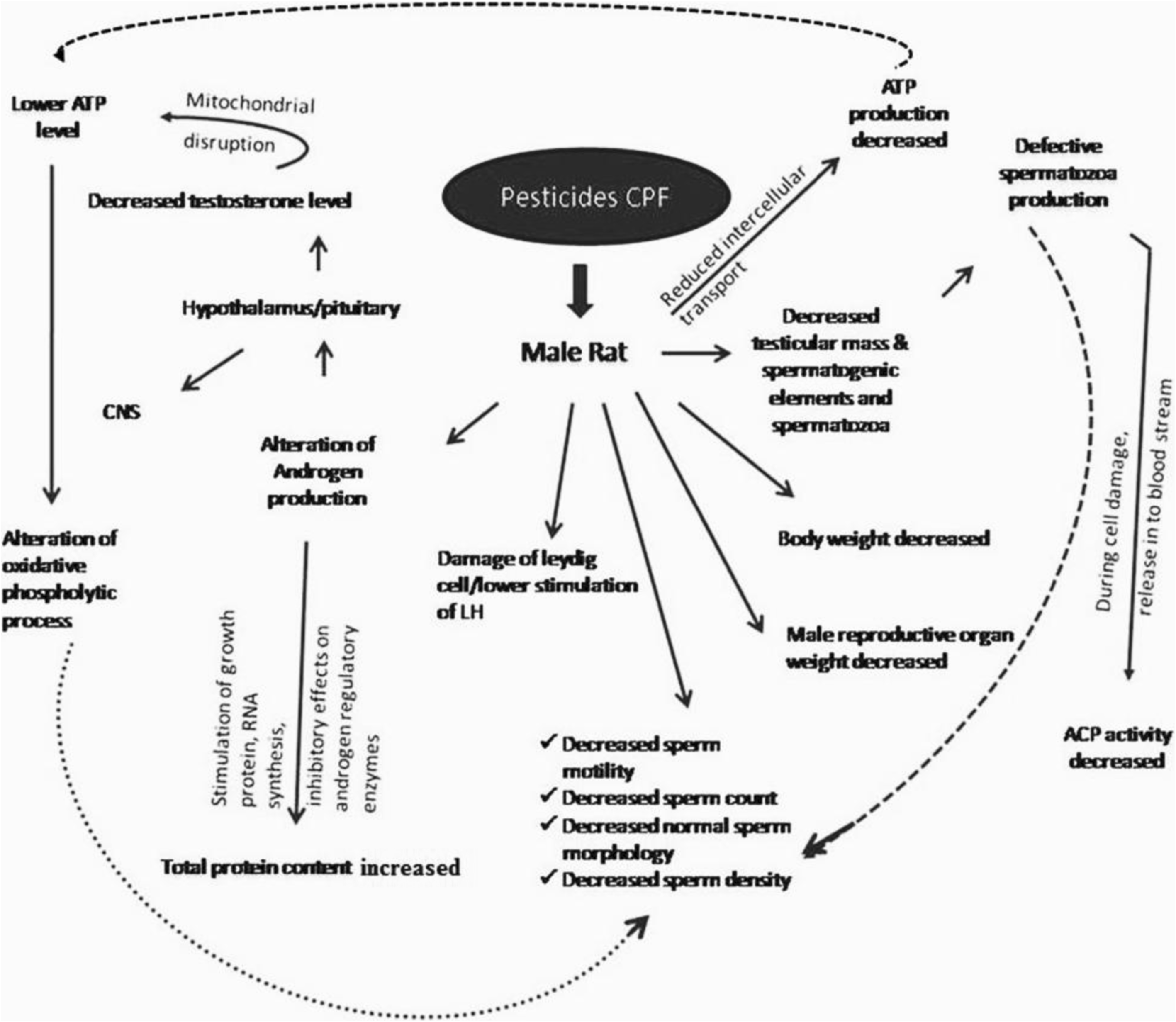

Supplement: Supplementary file 5 — Authors’ original file for figure 5 [file 40064_2013_598_MOESM5_ESM.tif]

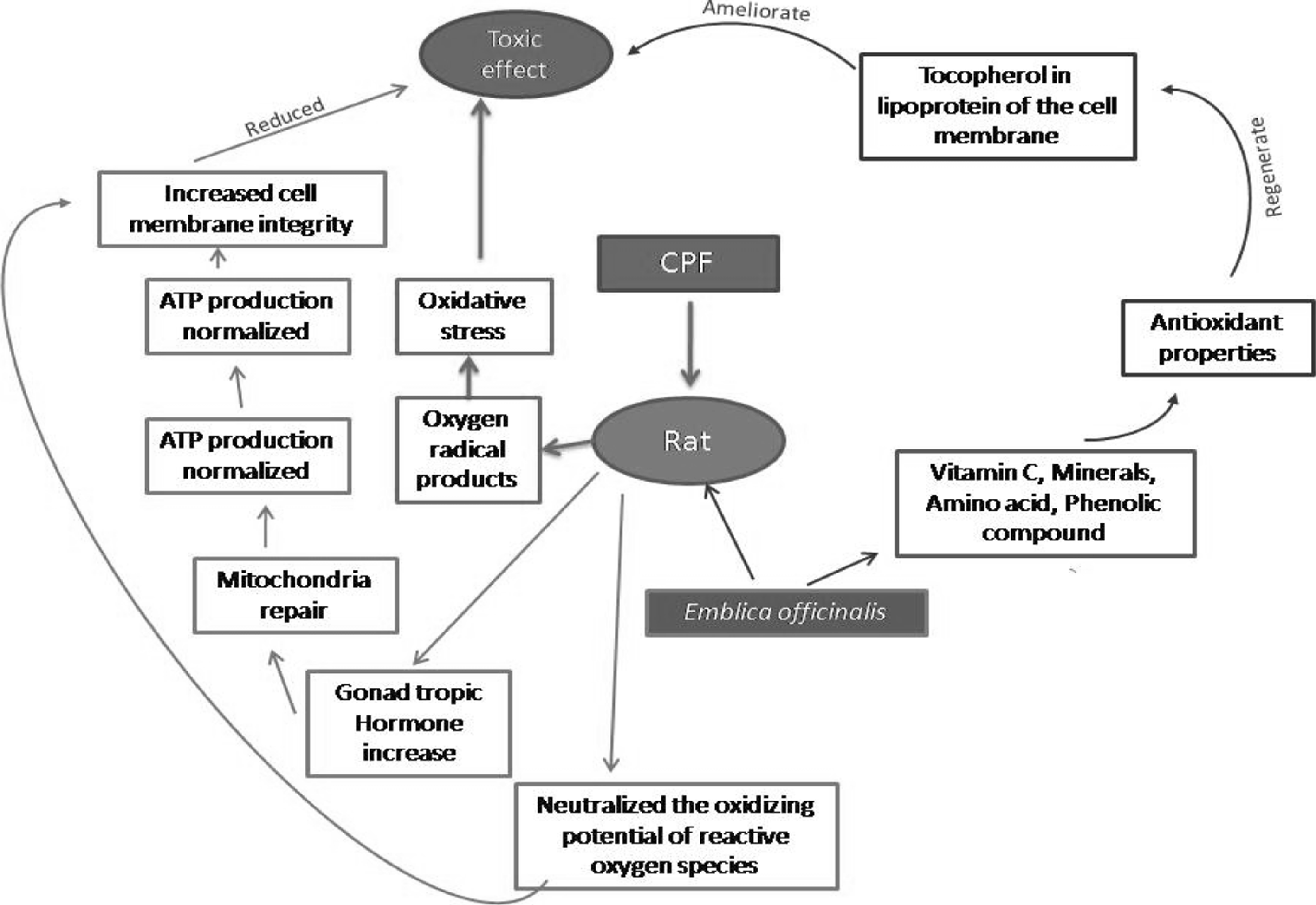

Supplement: Supplementary file 6 — Authors’ original file for figure 6 [file 40064_2013_598_MOESM6_ESM.tif]
