# Supplementary material for: Emblica officinalis Garten fruits extract ameliorates reproductive injury and oxidative testicular toxicity induced by chlorpyrifos in male rats
Source: Springerplus. 2013 Oct 17;2(1):541. doi: 10.1186/2193-1801-2-541 (PMC3824715; doi:10.1186/2193-1801-2-541)
Supplement: Supplementary file 8 — Authors’ original file for figure 8 [file 40064_2013_598_MOESM8_ESM.doc]

# Figure 6: The diagram showing the probable mechanism of remedial effects of Emblica against the pesticides treated tissue.

**
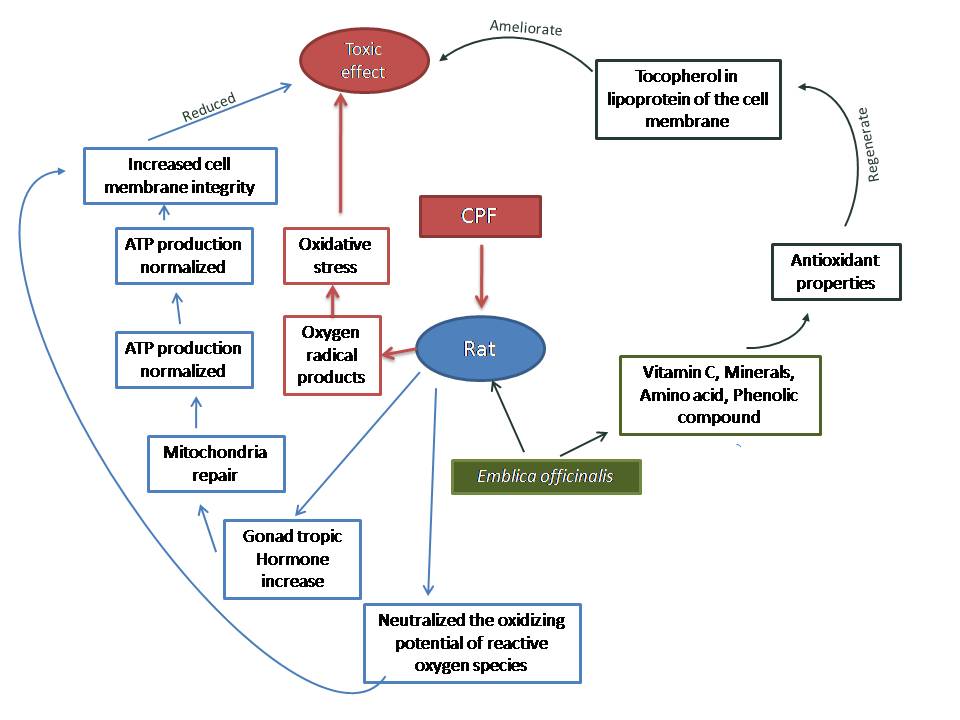
**
